# Actions speak louder than words: the case for responsible scientific activism in an era of planetary emergency

**DOI:** 10.1098/rsos.240411

**Published:** 2024-07-17

**Authors:** Tristram D. Wyatt, Charlie J. Gardner, Aaron Thierry

**Affiliations:** ^1^ Department of Biology, University of Oxford, Oxford, UK; ^2^ Centre for Biodiversity and Environmental Research, University College London, London, UK; ^3^ Durrell Institute of Conservation and Ecology, University of Kent, Canterbury, UK; ^4^ School of Social Sciences, Cardiff University, Cardiff, UK

**Keywords:** social movements, civil disobedience, ethics, climate, biodiversity, policy

## Abstract

The world's understanding of the climate and ecological crises rests on science. However, scientists' conventional methods of engagement, such as producing ever more data and findings, writing papers and giving advice to governments, have not been sufficiently effective at persuading politicians to act on the climate and ecological emergency. To date, governments’ decisions (such as continuing with vast subsidies for fossil fuels) clearly show that powerful vested interests have been much more influential than the amassed scientific knowledge and advice. We argue that in the face of this inaction, scientists can have the maximum amount of influence by lending their support to social movements pressing for action, joining as active participants and considering civil disobedience. Scientists seeking to halt continued environmental destruction also need to work through our institutions. Too many scientific organizations, from national academies of science to learned societies and universities, have not taken practical action on climate; for example, many still partner with fossil fuel and other compromised interests. We therefore also outline a vision for how scientists can reform our scientific institutions to become powerful agents for change.

## 1. Introduction

‘To know, and not to act, is not to know’ - Wang Yang-ming

**Figure 1.**
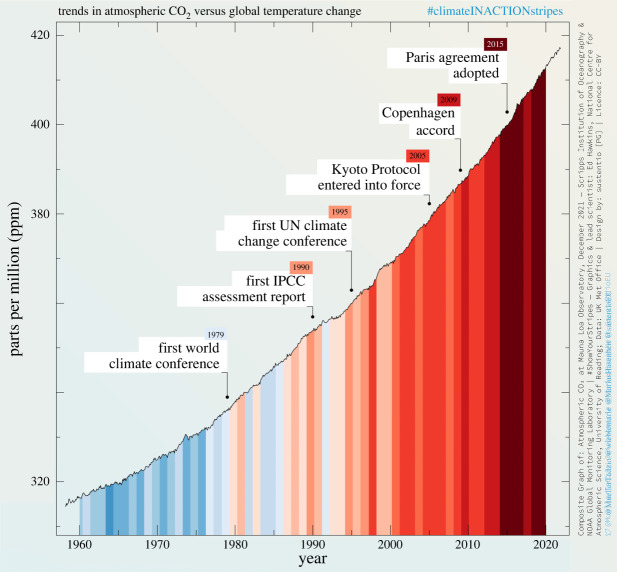
Carbon dioxide emissions continue to rise despite international agreements. Diagram credit: CC-BY @MuellerTadzio, @wiebkemarie, @MariusHasenheit, @sustentioEU [PG] https://sustentio.com/2022/climateinactionstripes-virale-klimakommunikation.

After decades of meticulous scientific inquiry, there is now an overwhelming consensus that to address the interlinked climate and nature crisis, countries must urgently phase out the fossil fuel economy and stop the destruction of biodiversity long before 2050 [1,2]. However, despite the establishment of the first global frameworks on climate change and biodiversity back in 1992, and near-annual Conventions of the Parties (COPs) ever since, atmospheric greenhouse gas concentrations continue to rise inexorably (figure 1) and biodiversity loss continues apace [3]. Some 60% of all greenhouse gas emissions have been produced since the first Intergovernmental Panel on Climate Change (IPCC) report [4], while 2023 was both the hottest year on record and the year with the highest ever greenhouse gas emissions [5,6]. Decades of scientific activity, including vast numbers of peer-reviewed publications, reports, conferences, participation in COPs and advisory roles in government, have not stimulated adequate progress. There is a conventional assumption that scientists can change the world through teaching and research, but these have simply not been effective in bringing about the radically transformative changes that science has demonstrated are required [4,7,8].

If scientists want to be more than chroniclers of a preventable tragedy, and to see science-informed policies enacted, we therefore need to seriously reexamine our theories of change. Rather than confining ourselves to inadequately effective methods, we should learn from successful movements for social change and adopt more effective approaches, including civil disobedience.

In this Commentary, we reflect on the evidence base of how scientists can best contribute to rapid, transformative societal change and supplement this with our own experiences as members of Scientists for Extinction Rebellion (www.scientistsforxr.earth) in the UK and Scientists Rebellion (scientistrebellion.org) which is active in more than 30 countries [9,10]. Both are groups of scientists who use peaceful non-violent civil disobedience, among other tactics, to push for emergency action on the climate and nature crisis. While this commentary will focus on fossil fuels and climate change, there are similar stories with the challenges to halting the decline in biodiversity, the other part of the planetary emergency.

## 2. Political capture by fossil fuel and wider corporate interests

For too long, scientists have laboured under the default assumption that our role was limited to carrying out and publishing research, in the apparent expectation that decision-makers would use the information to make rational decisions in the interest of their citizens [11]. But as governments' decisions supporting the continued expansion of fossil fuels clearly show, powerful vested interests have been much more influential than this scientific knowledge [12,13], with the result that the scientific community's collective warnings are overridden in decision-making.

Historically, fossil fuel companies have been the most profitable companies ever and there is clear and growing evidence that they have used this wealth to influence politics to delay action on climate change and continue with business-as-usual [14]. Some of the influence is direct; the fossil fuel industry spends more than $120 million each year on lobbying USA politicians [15], and £8.4 million has been donated from climate sceptics and fossil fuel interests to the ruling UK Conservative Party since the last election [16]. Perhaps equally important is the opaque funding by fossil fuel interests and wealthy neoliberal fundamentalists of ‘think tanks’, such as the Cato Institute in the USA and the Institute of Economic Affairs in the UK, which then lobby government and act as advisors [17]. Key to this influence over politicians and the wider public is the role played by a media owned by a small number of highly influential and wealthy individuals and groups, which uphold continued business-as-usual and regularly platform voices linked to the industry-funded think tanks. For example, in the UK, DeSmog identified 10 authors in the Daily Telegraph newspaper with links to the UK's most prominent climate science denial group, the Global Warming Policy Foundation think tank [18].

There has therefore been a deliberate, sustained and successful effort to undermine science- and evidence-based policy making. The fossil fuel industry's strategy has been to deny responsibility, spread doubt and delay effective action on climate change. A substantial evidence base now shows how they have adopted techniques used successfully by the tobacco industry, involving some of the same public relations (PR) strategists [19–21]. Such PR agencies are a critical, less examined organizational influence on climate policy, often working behind the scenes to shape public opinion on climate [22]. Fossil fuel companies continue to mislead the public by forefronting their investments in renewable energies [23] even though in 2022 these amounted to less than three percent of their investments in traditional fossil fuel exploration and production [24]. This misrepresentation is a practice increasingly termed ‘greenwashing’.

Powerful vested interests similarly delay action on threats to biodiversity [3] and the processes of political capture follow the pattern of fossil fuel interests [19]. For example, the ‘big meat’ livestock sector, directly and indirectly responsible for deforestation as well as greenhouse gas emissions, uses think tanks as one route to influencing legislators and public opinion [25,26].

## 3. Scientists as climate activists

The esteemed climate scientist Michael Mann [27] persuasively argued a decade ago that scientists must be outspoken about the risks we face and the need for an urgent societal response; succinctly put, ‘*If you see something, say something*’. We agree, but insist that given the gravity of our situation, and the political reasons for the current state of inaction described in the section above, scientists must go further and put our words into action. As the conventional activities of scientists have not been successful in catalysing change that is rapid and transformative enough to address the crises we face, our approach to effecting change needs to be reconsidered [7,28].

It is now increasingly recognized that strong social movements, rather than governments or the private sector, offer the greatest potential to catalyse the transformative changes required [4], with even the latest IPCC synthesis of climate mitigation stating with ‘high confidence’ that ‘collective action as part of social or lifestyle movements underpins system change’ [29]. The public pressure for change needs to be strong enough to counter the power of the fossil fuel lobby and the inertia of current systems. How can we help, as scientists, to build momentum towards social tipping points where we collectively demand interventions such as removing fossil-fuel subsidies, countering the power and influence of fossil fuel interests, pushing for healthier and more sustainable economy, incentivizing decarbonized energy generation, building carbon-neutral cities and divesting from assets linked to fossil fuels [30,31]?

We can be inspired by and learn from the history of peaceful civil disobedience (civil resistance), from the Suffragettes, Mahatma Gandhi and the non-violent campaign for Indian independence, Rosa Parks and the Montgomery bus boycott organized by Martin Luther King Jr., and which have been instrumental in gaining votes for women, independence for India, civil rights, and LGBT rights [32].

**Figure 2.**
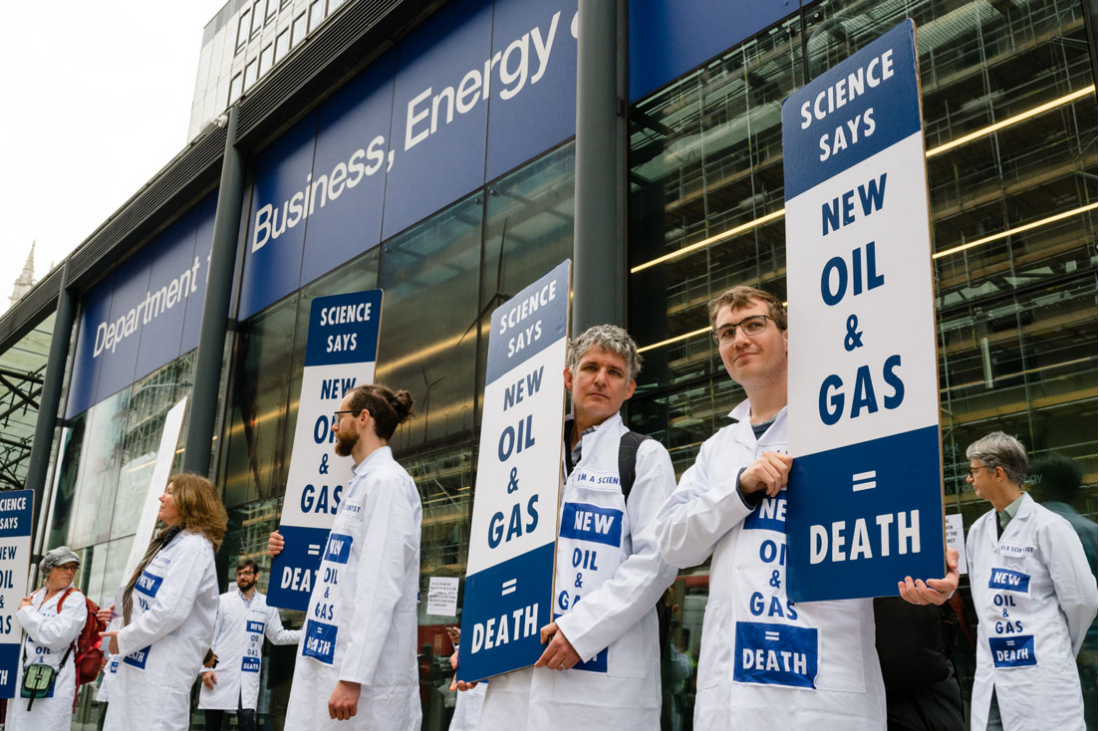
Scientists from Scientists for Extinction Rebellion protesting new oil and gas licensing outside of the Department for Business, Energy and Industrial Strategy, London. Photo credit: Andrea Domeniconi.

One aim of civil disobedience is to cut through the societal silence around climate change, to influence politicians and the public. To be ‘news-worthy’, protest actions usually require a disruptive, provocative or shocking element [33]. The renowned climate scientist and former director of the NASA Goddard Institute for Space Studies, James Hansen, drew international media attention when he was among 140 activists arrested outside the White House as part of a wave of protests that successfully blocked the Keystone XL pipeline from being built [34]. In April 2022, a few days after the latest IPCC WG3 report was published, Scientists for Extinction Rebellion targeted the UK government department responsible for fossil fuel policy to protest the government's decision to expand new oil and gas fields in the North Sea, against its own declared policy and advice from its own scientific advisors. Scientists glued enlarged title pages of scientific papers onto the glass of the building. Nine scientists were arrested and charged. This achieved front page coverage in one UK national newspaper and much more coverage besides, online and in print [35,36] (figure 2).

Scientists can give credibility to climate change campaigns firstly by our visible support. Wearing white laboratory coats, as a uniform to make us easily recognizable as scientists, has emerged as a tradition for scientists at protests (figure 2). This started with the 2017 March for Science in the USA. Our visible presence is particularly valuable for civil society movements, as scientists along with doctors are among the most trusted people in society, worldwide [37] and most of the public want researchers to get more involved in policy making [38].

Another aim of nonviolent civil disobedience is to create pressure on the government or authorities by disrupting the smooth functioning of society, for example by blocking infrastructure or entrances to key buildings, or interrupting meetings or cultural events [39]. There is an ongoing discussion across the environmental movement about the most effective targets for disruptive action. Some scientists have focused their direct action on symbolic high-carbon industries. For example, members of Scientist Rebellion joined other community groups to block private jets at airfields in Europe and the USA [40], while the group subsequently blocked a coal train in the Port of Amsterdam [41]. Alternatively, in other actions scientists have focused their civil disobedience on decision makers, for example taking part in actions at the offices of governmental, scientific and corporate institutions, perhaps most dramatically in Spain, where scientists threw buckets of fake blood on the steps of the Spanish Parliament [42].

On the basis of the historical evidence, we believe that climate protest including collective direct action and civil disobedience may be the most effective way of catalysing the changes the world so desperately needs (cf. [43]). Having said that, the range of approaches and tactics used by scientist activists and others across the climate movement belies the fact that our situation is unprecedented, and we lack much of an evidence base when it comes to what works and in what contexts. More research to guide the strategic development of the climate movement and enhance its effectiveness is an urgent priority, and the varied nature of activist approaches provides opportunities for action research and even experimentation in collaboration with activist groups. Growing numbers of academics are now pivoting their research focus towards these and other critical questions more pertinent to the emergency situation we face than the fields in which they established their careers (a particularly notable case being the neuroscientist Adam Aron [44]). Many others, though not engaging in nonviolent civil disobedience, are joining campaigning organizations such as Scientists for a Future, Researchers Desk, Union of Concerned Scientists, Science for the People and Scientists for Global Responsibility. Scientists can choose a level and type of involvement that is appropriate to their personal circumstances, privilege, skills and interests.

## 4. What can scientists do as scientists?

While scientists have power to accelerate change by acting as individuals within social movements, that power would be magnified if scientific institutions themselves were to lend their weight behind those movements. Scientists therefore need to campaign within their own institutions, such as campaigning for financial divestment and cutting research ties to fossil fuel companies [45]. British universities received more than £147 m in research funding and donations from fossil fuel corporations over the 7 years since 2016 [46]. The University of Exeter, despite being a leading centre for climate research in the UK, is a particularly egregious case having signed a £14.7 million deal with Shell in 2022 [47]. Fossil fuel funding also flows into universities in the rest of Europe and the USA [48,49]. This is despite evidence that, like tobacco sponsorship before it, fossil fuel funded research, whether by design or unwittingly, tends to produce results more favourable to their sponsors, which can be seen as part of ‘greenwashing’ and supporting delays to ending the fossil fuel economy (see e.g. [50,51]).

We need to redouble our efforts to campaign for fossil free research in our universities. This can include non-violent civil disobedience; for example, the Schlumberger-Out campaign is applying pressure on Cambridge University, which has an institute named after a company (SLB) providing scientific services for oil and gas exploration [52]. At the same time, we should be arguing for a ‘just transition’ in how research is funded; Earth sciences and chemical engineering departments, for example, have relied heavily on fossil fuel companies for research funds. Some well known scientific publishers that we rely on also directly profit from supporting fossil fuel exploration and production [53].

Too many scientific organizations, from national academies of science to subject-specific societies, are not taking public actions appropriate to the scale of the climate crisis and threats to biodiversity. Individuals in such institutions may tell us that, despite the lack of hard hitting public statements, they are working hard behind the scenes. However, the absence of any break in the rise of emissions (figure 1) and similar data showing a continuing loss of biodiversity shows that their quiet advocacy has not been effective.

The subject-area academic societies that many of us belong to should be a focus, as these could give a collective voice to our individual concerns. Scientists Peter Kalmus and Rose Abramoff without permission briefly held up a banner reading ‘Out of the lab & into the streets' at the start of a session of the Annual Conference of the American Geophysical Union (AGU) in 2022. The sanctions applied to Kalmus and Abramoff by the AGU in response were reversed when thousands of scientists from around the world, including James Hansen, wrote in support [54]. Thus a simple act of disobedience led to a lot of much needed scrutiny and debate. The following year, the AGU Conference had a whole session devoted to scientist activism, theory and practice, in part organized by Kalmus and Abramoff, which demonstrates the power of their action in rapidly changing the culture of the AGU. However, there remains an ongoing debate within the AGU about taking fossil fuel sponsorship [55]. In the field of ecology, the British Ecological Society (BES) invited members to run a workshop on Scientists as Activists, using their experience in Scientists for Extinction Rebellion and Scientist Rebellion, later given the cover story in the BES's The Niche [56]. The growing prominence of such discussions is an encouraging sign that scientific organizations are increasingly reflecting on the ways they can contribute effectively to processes for social change.

However, it seems that as yet no country's national academy of science has taken a leading role in campaigning for a transition from fossil fuels. Scientists should perhaps be more vocally challenging our scientific leadership, as Scientist Rebellion's founders did in 2020 with a symbolic paint spilling action against the United Kingdom's Royal Society [57]. Individual Fellows of the Royal Society have expressed disappointment with, for example, the lack of progress at COP 27 [58], but the Royal Society has singularly failed to publicly denounce current UK government policy to licence new oil and gas. A letter from more than 1000 UK academics, including many Fellows of the Royal Society, has demanded an unambiguous statement from the Royal Society about the culpability of the fossil fuel industry in driving the climate crisis [59,60]. The Royal Society's hesitancy, equivocation and silence to date contrasts with the stance of António Guterrez, Secretary General of the United Nations, who clearly states that ‘investing in new fossil fuels infrastructure is moral and economic madness' and that ‘the truly dangerous radicals are the countries that are increasing the production of fossil fuels’ [61].

## 5. What holds scientists back from activism?

Many scientists are already partaking in some degree of activism; a survey of IPCC authors found that a quarter attended demonstrations as well as participating in other activities [62]. Another more recent and larger survey of scientists found that 29% of respondents have engaged in climate advocacy, 23% had joined legal protests and 10% in civil disobedience noting that ‘*climate researchers reported engaging, on average, in twice as many civic actions as non-climate researchers*' [63]. Why are other scientists reluctant to get more involved in activism on climate and other issues? Among a survey of almost 2000 UK academics across all subjects, a majority cited lack of time, due to demands on staff in modern university life [64]. Many were also unsure what the best things to do would be, but at same time were in principle behind the idea of activism and being actively involved in helping to create change. In another survey, of more than 300 respondents, scientists who did not see a conflict between being an activist and scientist were more likely to be involved in activism [65].

Another key barrier to a scientist becoming an activist and in particular becoming a protestor, is not knowing someone who is already involved in climate activism [63]. The possible lack of support from the university administration was another reason for a reluctance of some academics to protest [64]. Many will perhaps be surprised to learn that ‘there is a very high level of peer support for climate advocacy by other researchers’ [64], suggesting that researchers are likely underestimating the support they would receive if they were more outspoken. Senior academics can help by setting an example, and ensuring that their universities' policies support staff involved in activism including peaceful civil disobedience [7,66].

Some scientists fear that speaking out as an advocate will reduce our credibility as objective observers. However, the opposite may in fact be the case: some research with the general public suggests that advocacy and climate activism can increase credibility, paradoxically, in part because the public expects us to act as we are well informed; they will be surprised if we didn't [67–69]. Not taking action risks sending a signal that perhaps the climate and ecological emergency isn't as bad as our warnings are seeking to convey (i.e. if scientists are acting calmly and following business as usual, surely things can't be that serious). As the manifesto of Scientist Rebellion says, ‘*Unless those best placed to understand behave as if this is an emergency, we cannot expect the public to do so*’ [70].

Advocacy by scientists has a proud history [71]. For example, the Nobel laureate Sherry Rowland actively campaigned for control of CFCs that his research had identified as a danger to the ozone layer. Some scientific colleagues were not supportive, but he famously said ‘*What's the use of having developed a science well enough to make predictions if, in the end, all we're willing to do is stand around and wait for them to come true?*’ (cited in [72]). There is a good case that scientists have both an obligation to speak out and that it is ethical to do so [72,73]. There have been many political campaigns for public health and social justice that have been taken up by health professionals, from Florence Nightingale onwards [74] and recently nurses and doctors were very active on behalf of climate change advocacy, for example in the 2022 Australian federal elections [75].

Scientific activist organizations are recognizing that while in many instances academics do occupy a relatively high level of safety and privilege, the risks of getting involved in direct action are not the same for everyone, whether because of sex, class, race, and the precarious nature of employment for many academics [9]. We emphasize that activism and actions can come in many forms—from letter campaigns to teach-ins, occupations, boycotts and disruption of events, infrastructure and institutions. Many of the essential roles in protest actions are behind-the-scenes, far from the public glare or a police presence. We encourage scientists to contribute the skills and time they are able to, whether that's in organization, mobilization, wellbeing support, public outreach, press liaison, social media, arts and graphics, photography and live streaming, and protest liaison. Scientists have also given expert testimony on behalf of those on trial for peaceful climate protest actions. Given the respect that scientists hold, even the simple act of showing public support for climate activist groups is powerful, in the face of widespread media demonization and growing political and legal crackdowns on protest in many countries [76]. Scientists and scientific institutions must defend academic freedom, and that must include the right for scientists to participate in legitimate peaceful protest.

## 6. Growing a movement of scientist activists

‘Anything else you're interested in is not going to happen if you can't breathe the air and drink the water. Don't sit this one out. Do something. You are by accident of fate alive at an absolutely critical moment in the history of our planet.’ - Carl Sagan

Although there are now thousands of scientist activists in dozens of countries around the world, it is still a small proportion of the scientific community, perhaps in the low thousands, they have helped generate a lot of press and public attention towards the planetary emergency and started to shift norms of academic behaviour. However, if these impacts are to grow to a scale appropriate for addressing a planetary emergency, we will need enormous growth in numbers. We need to be recruiting many more scientists, to take on the many different kinds of roles in activism. Key to our effectiveness will be working together with other social movements, in all their diversity [71].

It is noticeable that our events on climate change activism at scientific conferences tend to be fringe meetings which may mostly attract those who are already sympathetic to the idea of activism. Given the central importance to all scientific disciplines of understanding the threats to life on Earth, and what we can do in response, such sessions would be much more fitting as plenaries for all delegates. If our scientific organizations recognize that we are in a climate and ecological emergency, we must also recognize that emergency times call for emergency measures and break with the status quo. It is not appropriate to be comfortable with the default simply because that's the way it's always been.

Although activism does require scientists to step outside of our comfort zones and breach long-held academic norms, we face a planetary emergency and scientists need to show courage and be bolder in taking principled stands. We hope that this commentary will help serve as a guide by reassuring colleagues that the path to scientific activism is a reasonable and rewarding one to embark on. Scientists can also learn from the ways that doctors (in many ways a notably conservative profession) have been so much more vocal on climate change, including those in more senior positions. For example, the editors of more than 200 health journals recently published a simultaneous editorial arguing that it was ‘Time to treat the climate and nature crisis as one indivisible global health emergency’ (Abbasi *et al*., [1]). It is not clear to us why so many senior scientists have so far, by comparison, been silent.

Einstein said ‘*Those with the privilege to know have a duty to act*’. We **know** that if we don't act now we face a rapidly worsening environmental and societal disaster. We **know** that our observations and warnings alone haven't worked. We **know** we are up against powerful vested interests. We **know** that social movements are key to social change and that we have to build democratic counter power. Given what we know, we must help support, defend and participate in those movements pushing for transformative change! Join us!

## Data Availability

This article has no additional data.
